# Positioning error of custom 3D-printed surgical guides for the radius: influence of fitting location and guide design

**DOI:** 10.1007/s11548-017-1682-6

**Published:** 2017-11-06

**Authors:** G. Caiti, J. G. G. Dobbe, G. J. Strijkers, S. D. Strackee, G. J. Streekstra

**Affiliations:** 1 Department of Biomedical Engineering and Physics, Academic Medical Center, University of Amsterdam, Amsterdam, The Netherlands; 2 Department of Plastic, Reconstructive and Hand Surgery, Academic Medical Center, University of Amsterdam, Amsterdam, The Netherlands

**Keywords:** Radius osteotomy, Patient-specific surgical guides, 3D-printing, Computer-assisted orthopedic surgery

## Abstract

**Purpose:**

Utilization of 3D-printed patient-specific surgical guides is a promising navigation approach for orthopedic surgery. However, navigation errors can arise if the guide is not correctly positioned at the planned bone location, compromising the surgical outcome. Quantitative measurements of guide positioning errors are rarely reported and have never been related to guide design and underlying bone anatomy. In this study, the positioning accuracy of a standard and an extended guide design with lateral extension is evaluated at different fitting locations (distal, mid-shaft and proximal) on the volar side of the radius.

**Methods:**

Four operators placed the surgical guides on 3D-printed radius models obtained from the CT scans of six patients. For each radius model, every operator positioned two guide designs on the three fitting locations. The residual positioning error was quantified with a CT-based image analysis method in terms of the mean target registration error (mTRE), total translation error ($\Delta T$) and total rotation error ($\Delta R$) by comparing the actual guide position with the preoperatively planned position. Three generalized linear regression models were constructed to evaluate if the fitting location and the guide design affected mTRE, $\Delta T$ and $\Delta R$.

**Results:**

mTRE, $\Delta T$ and $\Delta R$ were significantly higher for mid-shaft guides ($p=0.0001,\,p= 0.0001\,\hbox {and} p=0.001$) compared to distal guides. The guide extension significantly improved the target registration and translational accuracy in all the volar radius locations ($p=0.001$). However, in the mid-shaft region, the guide extension yielded an increased total rotational error ($p= 0.0001$).

**Conclusion:**

Our study demonstrates that positioning accuracy depends on the fitting location and on the guide design. In distal and proximal radial regions, the accuracy of guides with lateral extension is higher than standard guides and is therefore recommended for future use.

## Introduction

In the last decade, computer-assisted three-dimensional (3D) preoperative planning has been adopted in an increasing number of orthopedic, oral and maxillofacial surgical procedures [1–6]. Three-dimensional preoperative measurements for surgical planning, based on virtual bone models reconstructed from a computed tomography (CT) scan of a patient, are more reliable than measurements from traditional plain radiographs, because they do not suffer from overprojections and hidden rotations about the longitudinal axis of long bones [7, 8]. Furthermore, with 3D planning software, surgical cutting planes and drilling trajectories can be accurately planned on the virtual bone models in six degrees of freedom.

An accurate navigation technique is essential to transfer the 3D preoperative surgical planning to the patient during surgery. Patient-specific 3D-printed cutting, drilling or reduction guides are used for this purpose. These guides are customized molds that fit onto the bone of the patient, featuring cutting slits and drilling holes to directly position the surgical tools as planned. Patient-specific surgical guides have shown to reduce surgery time, radiation exposure and are perceived as being easier to use than marker-based navigation systems by surgeons [1, 9]. However, navigation errors arise when malpositioning the customized guide on the corresponding bone. Malpositioning is likely to occur, as also demonstrated by Van den Broeck et al. [10] who experimentally investigated the stability of custom guides by applying an external force and found that errors introduced by the initial manual positioning of the guide sometimes had a major effect on the guide stability. Since a guide positioning error implies an error in the therapeutic action (e.g., drilling, cutting), inaccurate guide positioning can compromise the overall outcome of the navigated surgery.

The positioning accuracy of the patient-specific surgical guide depends on characteristic bone prominences or surface anchors, covered by the guide. For instance, distal, mid-shaft and proximal regions of the radius bone in dorsal and volar aspects greatly differ in shape and distribution of surface anchors. Consequently, guides of the same size are likely to perform differently at these locations.

Another factor directly related to the guide positioning accuracy is the amount of bone surface covered by the guide. Most commonly used customized guides are designed to sit on top of the volar or dorsal side of the radius [9, 11–14]. In this report, we introduce an alternative guide design with an additional lateral extension to encapsulate the outline on the side of the bone, which may improve the accuracy of positioning.

The accuracy of guide positioning on the radius has never been quantitatively investigated in relation to the fitting location and guide design. In this laboratory study, we used a CT-based methodology to measure the error of positioning two 3D-printed custom guide designs (standard and extended guides) at different fitting locations (distal, mid-shaft and proximal) on the volar side of the radius. We hypothesize that the guide positioning errors are influenced by the fitting location and by the guide design.

## Materials and methods

The description of planning and analysis methods as well as experiments performed in this study is organized in six parts, labeled A to F:(A) the CT-based design and manufacturing of the custom guides and bone models; (B) the evaluation of methodological errors due to the 3D printing of the physical models used for our experimental evaluation; (C) the study performed to evaluate the accuracy of custom guide positioning; (D) the CT-based technique to measure the positioning errors; (E) the experiment carried out to evaluate the accuracy of the CT- based methodology for quantifying the positioning errors; and (F) the statistical analysis. Parts A-C-D-F are directly related to the guide positioning accuracy evaluation. Parts B and E refer to preliminary experiments conducted to evaluate the methodological accuracy. These parts are detailed below.

**Fig. 1:**
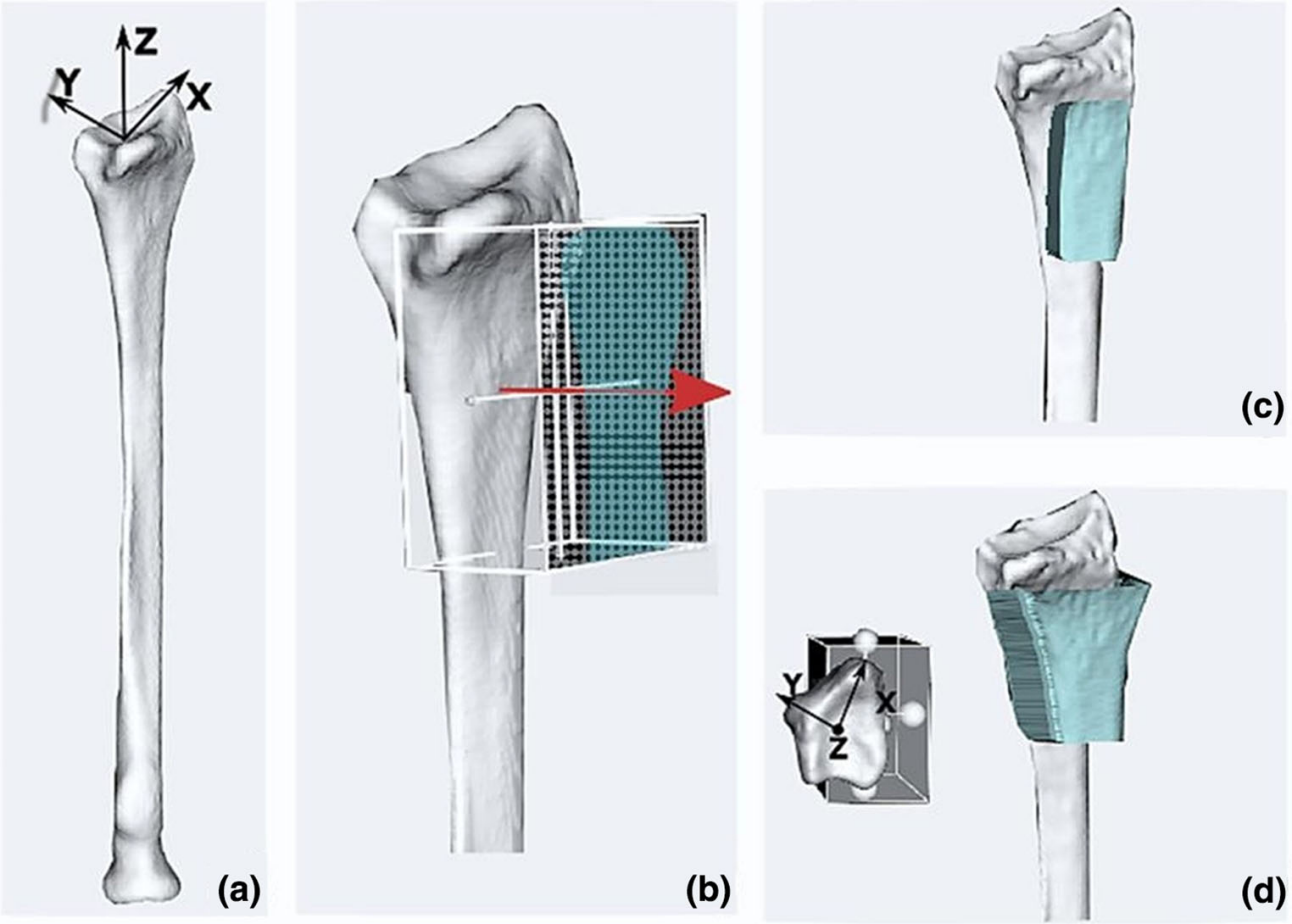
Patient-specific guide design **a** 3D virtual model of the radius; **b** Virtual box enclosing the polygon surface showing a regular grid of points (2D projection image) on the box face used for projection, points projecting onto the bone surface are highlighted. The arrow points toward the direction of the extrusion; **c** Extrusion of the selected volar radius surface generates a standard guide **d** Points alongside the bone are projected toward the opposite side of the virtual box that is positioned halfway through the virtual bone in the coronal plane (left). Extrusion of the projected surface generates an extended guide (right)

### Computer-aided design and 3D printing of models and guides

Six 3D virtual bone models of healthy right adult radiuses were obtained from CT scans acquired for a previous study [15]. These healthy radiuses mimic the worst-case scenario for guide positioning, since custom guides for the radius are generally applied in corrective osteotomy surgeries to deformed bones [7, 14, 16] showing more prominent surface anchors than healthy bones. Bone segmentation was performed as described in [7]. In brief, with custom-made software, each radius was first segmented via a level-set algorithm initialized via threshold-connected region growing and binary filling. Subsequently, a polygonal description of the segmented bone was extracted at the zero level of the level-set segmentation (Fig. 1a).With the same software, we first selected the target surface for creating custom guides by interactively sizing and positioning a virtual box enclosing the bone polygon target surface. Then, a regular grid of points (2D binary projection image) was created on one face of the box (Fig. 1b). The binary 2D projection image was then eroded to omit grid points that projected onto the edge of the bone. After smoothing the contour of the 2D image with a binary opening operator, the remaining points of the 2D image were projected onto the polygon surface to create the footprint of the custom guide. The footprint was subsequently extruded by 20 mm in a direction interactively chosen by the user to generate the standard guide. In order to create extended guides, encapsulating the outline on the side of the bone, the same procedure was used with two differences: (1) erosion was replaced by dilation to add points that extend beyond the contours of the bone; (2) the additional points alongside the bone were projected onto the opposite face of the box, which was manually positioned to enclose approximately 50% of the selected bone volume in the coronal plane (Fig. 1d).

**Fig. 2:**
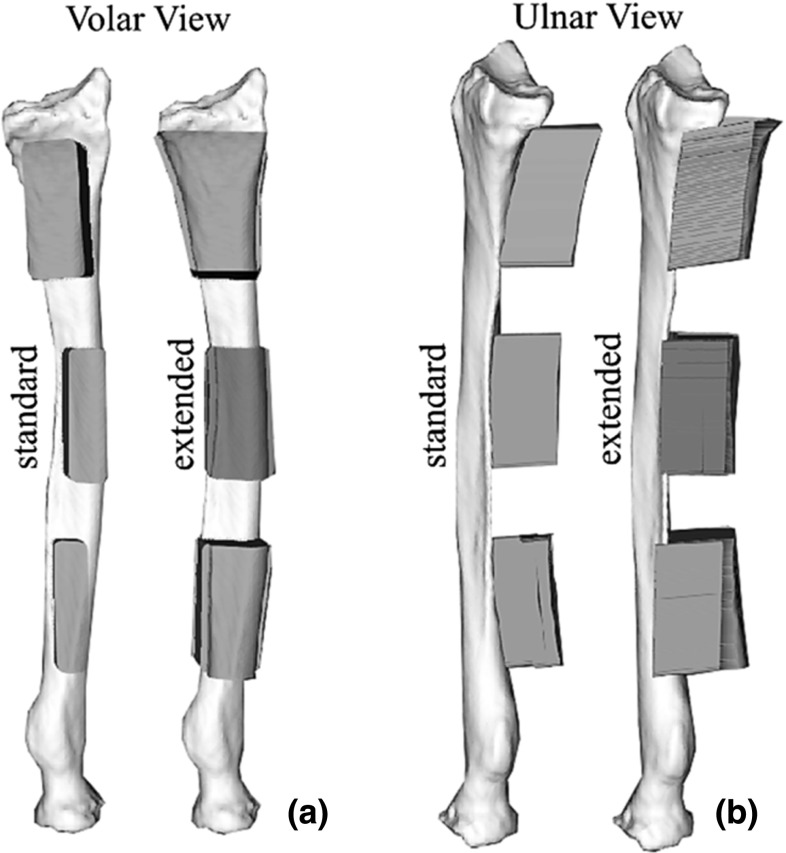
Same radius with standard and extended guides fitting the distal, mid-shaft and proximal regions. **a** Volar view **b** ulnar view

Following the described methodology, we designed, for each radius, two sets of three patient-specific guides (Fig. 2). The three guides in each set respectively fit the distal, mid-shaft and proximal surfaces on the volar aspect of the radius. The distinction between distal, mid-shaft and proximal parts of long bones is based on an equal one-third division of the length of the radius [17]. The first set included standard guides, and the second set included extended guides. The length of the guides was chosen 20% of the length of the radius in both sets. We also 3D-printed reference models of each radius with guides attached in the planned positions. These reference models were made for each of the guide designs. Finally, six radius models, $6\times 2$ reference models with standard and extended guides attached, and the two sets of $6\times 3$ guides were 3D printed in Polycarbonate-ISO (PC-ISO), with a Fortus 450 mc fused deposition modeling (FDM) printer (Stratasys, Eden Prairie, Minnesota, USA). The printing accuracy was of $\pm \,0.127$ mm in all directions.

### Agreement between 3D-printed and virtual model

In order to evaluate whether the original virtual bone model was correctly represented by the 3D-printed model, we performed a preliminary CT-based experiment. We acquired a CT scan of 3D-printed bone model. A dedicated scanning protocol was used to acquire the CT scans (tube charge 500 mAs; tube voltage 120 kV; slice thickness 0.67 mm; voxel size $0.33\times 0.33\times 0.33\,\hbox {mm}^{3}$; and pitch 0.609). After segmentation, we aligned this bone model with the virtual bone that was originally used as a template for the 3D print by registration (described in “CT-based analysis of guide positioning errors” section). As a measure of distance between the virtual and the printed bone model, we determined the average point-to-point Hausdorff distance between the aligned 3D-reconstructed polygons and the printed bone model. Given two point sets $A=\left\{ {a_1 } \right. \ldots \left. {a_p } \right\}$ and $B=\left\{ {b_1 } \right. \ldots \left. {b_p } \right\}$, the Hausdorff distance is defined as $H\left( {A,B} \right) =\max \left( {h\left( {A,B} \right) ,h\left( {B,A} \right) } \right)$ where *h*(*A*, *B*) represents the maximum nearest-neighbor distance of the points in *A* to the points in *B*: $h\left( {A,B} \right) \quad h\left( {A,B} \right) =\mathop {\max }\nolimits _{a\in A} \mathop {\min }\nolimits _{b\in B} \Vert a-b\Vert$ [18].

### Data acquisition and study design

Four independent operators (two recently graduated medical doctors with 6–9 months of experience as interns in the plastic surgery department and two experienced surgeons) positioned the guides on the six 3D-printed bone models of healthy radiuses. In order to attach the guides to the radiuses, we used a solvable glue (Gluo Pen Duo, Tesa, Norderstedt, Germany) that washes out in cold water ($30^{\circ }$). Every operator first positioned the extended guides on each radius model. After at least one week, the same operator repeated the positioning for the sets of standard guides. A paper copy of the 3D planning, showing the planned guide positions, was provided to the operators for guidance, as in the case of real surgery. Every operator used the same bone models and guides to avoid possible variability introduced by 3D printing multiple copies of a bone.

### CT-based analysis of guide positioning errors

**Fig. 3:**
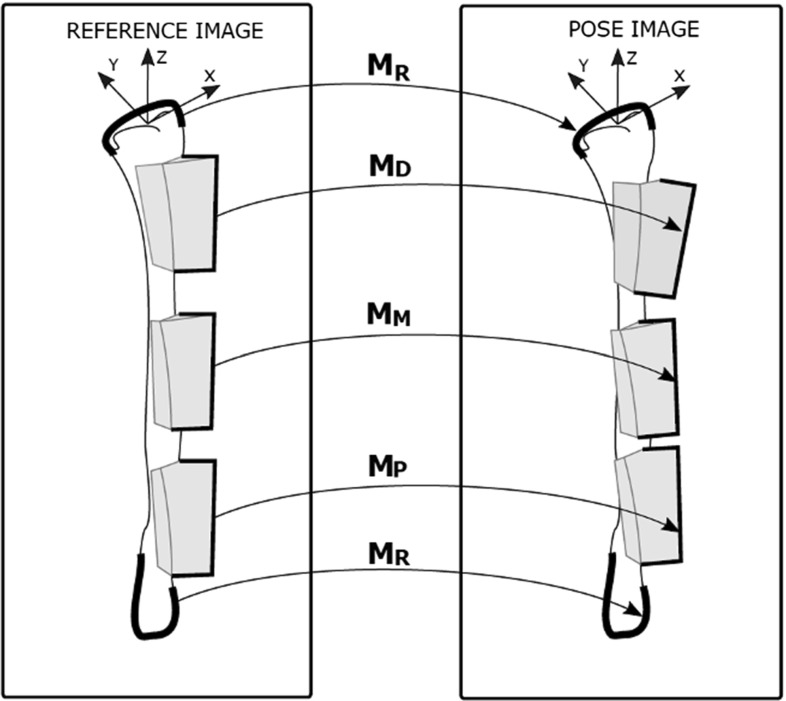
Alignment of reference bone (distal and proximal parts) and the selected portions (bold) of the three guides from the reference image for registration to each pose image

After guide positioning, we acquired CT scans of each radius models with the attached guides (pose images). For each of the six bone geometries and each of the two guide designs, we also acquired CT scans of the reference model with the guides printed in the planned positions (reference images). We were interested in the relative positioning error of each guide with respect to the reference radius. Since the radius itself was in a different position in each acquired image, we first needed to register each pose image with the reference image. The pose and the reference images were registered with a point-to-image intensity-based registration technique, as also described in [7]. In brief, in the reference image, we segmented the bone model with the guides attached in the planned position. For the segmentation, we adopted a Laplacian level-set growth algorithm initialized by the result of threshold-connected region growing. A polygonal description of the reference model was then extracted at the zero level of the level set. By sampling the gray values of the image along the inner and outer contour of the polygon, we obtained a double-contour polygon to be used for image registration. Then, we clipped and grouped the distal and the proximal part of this double-contour polygon, thus isolating the portions of the bone not covered by the guides. We also clipped the guides, excluding the points close to the bone surface. Each clipped subset of the double-contour (bone and guide) polygons was then registered to the pose image with a rigid point set-to-image registration procedure. The registration method used the Nelder–Mead downhill simplex optimizer with a six-parameter search space (three displacements and three rotations). The correlation coefficient was used as metric unit to quantify how well the gray-level points fit the reference image [7]. Registration of the distal and proximal segments of the bone to the pose image yielded the point-to-image registration matrix ($M_R$). The registration of the clipped portions of distal, mid-shaft and proximal guide objects to the pose image yielded the registration matrices ($M_D ,M_M ,M_P$). This enabled a calculation of the error matrices:

$$\begin{aligned} E_{D}= & {} (M_R^{-1} M_D )\\ E_{M}= & {} (M_R^{-1} M_M )\\ E_{P}= & {} (M_R^{-1} M_P ) \end{aligned}$$

which bring each guide from the planned to the actual position in the reference image (Fig. 3).

Each transformation matrix *E* is a $4\times 4$ homogeneous matrix that transforms column vectors and represents a translation of the guide’s centroid ($c_{\mathrm{x}}, c_{\mathrm{y}}, c_{\mathrm{z}}$) to the origin of the reference frame, three rotations about the axes of the coordinate system and finally a translation of the centroid back to its original position slightly altered by the translation error ($\Delta _{\mathrm{x}} , \Delta _{\mathrm{y}} , \Delta _{\mathrm{z}}$):

$$\begin{aligned} E= & {} T\left( {c_x +\Delta _x ,c_y +\Delta _y ,c_z +\Delta _z } \right) \\&\times R_z R_x R_y T(-c_{x,} -c_{y,} -c_z ) \end{aligned}$$

With *T* being a translation and $R_{x}, R_{y}, R_{z}$, the rotation matrices describing the rotations about the axes of the reference frame. Since the transformation matrix *E* and the original centroid position are known, the translation error can be calculated.

Therefore, each error matrix comprises three displacement errors ($\Delta _x , \Delta _y , \Delta _z$) and three rotation errors ( $\varDelta \varphi _x$, $\varDelta \varphi _y$, $\varDelta \varphi _z$). These positioning errors were subsequently expressed in terms of an anatomic coordinate system for the reference radius. The right-handed anatomic coordinate system is defined as follows: The *z*-axis is the principal axis of inertia of the bone polygon, the *x*-axis points toward the styloid process, and the *y*-axis is oriented perpendicular to *z* and *x* (Fig. 3).

**Fig. 4:**
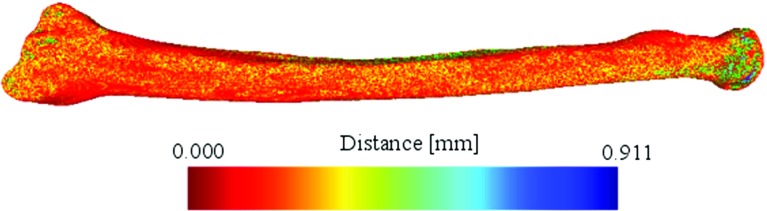
Hausdorff distance color map. Maximum distance is measured at the level of the radial head where none of the guides were positioned

**Fig. 5:**
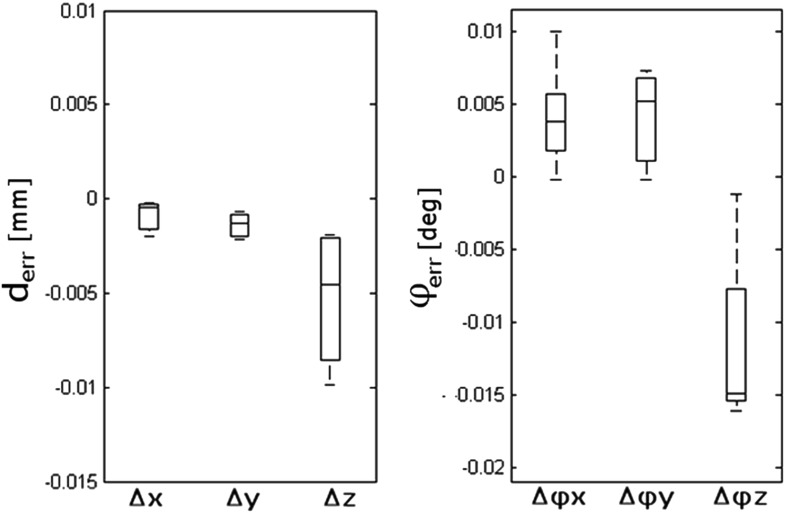
Box plot of errors occurring after CT-CT point set-to-image registration, when measuring the relative position of the guide with respect to the bone in the reference model

In order to combine all six error parameters into a single parameter, we also express the positioning errors in terms of the mean target registration error (mTRE) [19]. The TRE is defined as the distance between each surface point $\mathbf{p}_i$ on the clipped guide polygon contour in the reference image, which reflects the gold standard position, and the same point transformed with $M_R^{-1} M_{G}$(with *G* representing the fitting location: $\hbox {D}=\hbox {distal}$, $\hbox {M}=\hbox {mid-shaft}$, $p=\hbox {proximal}$) which represents the actual position of a guide. The average distance between the *n* points in a guide defines the mTRE [19]:

$$\begin{aligned} \hbox {mTRE}_G = \frac{1}{n}\mathop \sum \nolimits _{i=1}^n \Vert {\varvec{p}}_i -M_R^{-1} M_G {\varvec{p}}_i\Vert \end{aligned}$$

The mTRE was chosen as a single metric to establish which guide type generally performs better in terms of positioning accuracy. However, in order to interpret the guide positioning error in terms of translational and rotational errors, at the same time we report the total translation error $\Delta T$ and the total rotation error $\Delta R$, defined as in [20]:

$$\begin{aligned} \Delta T= & {} \sqrt{\left( {\Delta _x } \right) ^{2}+\left( {\Delta _y } \right) ^{2}+\left( {\Delta _z } \right) ^{2}}\\ \Delta R= & {} \sqrt{\left( {\Delta \varphi _x } \right) ^{2}+\left( {\Delta \varphi _y } \right) ^{2}+\left( {\Delta \varphi _z } \right) ^{2}}. \end{aligned}$$

### Evaluation of the CT-based technique to quantify positioning errors

Reproducibility of the CT-based technique to measure positioning errors (described in D) depends on manual initialization of the registration procedure, on the number of points present in the double-contour polygons and on the noise pattern of the images [7]. We investigated the accuracy and reproducibility of registration by CT scanning one 3D-printed reference model, with guides rigidly attached in the planned position, eight consecutive times without repositioning. We segmented the reference bone (distal and proximal radius parts) and the distal guide out of the first image and registered the selected parts to each of the remaining seven CT scans to find the positioning errors as described in “CT-based analysis of guide positioning error” section. Differences in the transformation parameters ($\varDelta x$, $\varDelta y$, $\varDelta z$, $\varDelta \varphi x$, $\varDelta \varphi y$, $\varDelta \varphi z$) provided the accuracy (mean error) and reproducibility of the method (standard deviation).

### Statistical Analysis

A total of 144 data points ($4\,\hbox {operators} \times 6\,\hbox {radiuses} \times 3\,\hbox {guides} \times 2\,\hbox {guide designs}$) were available for statistical analysis. After preliminary checking normality of data, three separate generalized linear models (normal probability distribution, identity link function) were constructed with SPSS (Version 24.0, SPSS Inc., Chicago, IL) for the multivariate analysis of the mean target registration error (mTRE), the total translation error ($\Delta T$) and the total rotation error ($\Delta R$). The categorical variables *Location* (which represents the position of the guide); *Extension*(which represents the guide design); and their combined effect $Location*Extension$ were used as predictors. $Location=Distal$ and $Extension=Yes$ were chosen as reference categories. In order to check if the different operators and the different bone geometries acted as confounding effects on the positioning error, the corresponding variables (*Operator*, *Geometry*) were also included in the full model as fixed effects. A Chi-square statistic index was used to assess the significance of the predictors included in the full model. Reduced models were then created by excluding nonsignificant predictors with a step-wise approach. A *p* value $<0.05$ was considered significant.

## Results

### Agreement between 3D-printed and virtual models

The accuracy of the 3D model, measured as the point-to-point Hausdorff distances between the original polygonal model and the scanned 3D-printed model, was ($\hbox {mean}\pm \hbox {SD}) = 0.170\,\pm \,0.095\,\hbox {mm}$. Maximum Hausdorff distance found was 0.911 mm occurring at the radial head on the dorsal side (Fig.4), where none of the guides were positioned.

### Evaluation of the CT-based technique to quantify positioning errors

The accuracy of the point set-to-image registration for the reference bone and the guide resulted in a translation error ($\hbox {mean}\pm \hbox {SD}) < 0.002\,\pm \,0.010\,\hbox {mm}$ and a rotation error ($\hbox {mean}\,\pm \,\hbox {SD}) < 0.013\,\pm \,0.010{^{\circ }}$. Figure 5 shows a box plot reporting median and Inter-Quartile Range (IQR) of the 6 DOF parameters.

**Table 1:** Summary of model effects in the exploratory models tested

	mTRE Model effects	Chi-square	*df*	*P* value
Model 1	*Extension*	10.555	1	0.001
	*Location*	12.381	2	0.002
	Location*Extension	3.725	2	0.155
	*Operator*	6.447	3	0.092
	*Geometry*	4.59	5	0.468
Model 2	*Extension*	10.734	1	0.001
	*Location*	12.656	2	0.002
	*Operator*	3.301	2	0.100
	Location*Extension	6.243	3	0.192
Model 3	*Extension*	11.022	1	0.001
	*Location*	13.058	2	0.001
	*Operator*	6.099	3	0.107
Model 4	*Extension*	10.562	1	0.001
	*Location*	12.513	2	0.002

Model 4 is the final model for mTRE

### Effect of fitting location and guide design on guide positioning errors

In the full statistical model (Model 1, Table 1), the variables $Location, Extension, Location*Extension, Operator$ and *Geometry* were included to evaluate their joint effect on the mTRE. Since *Geometry* had the least significant effect in the full model, it was excluded in the first reduced model (Model 2). In the same way, the variables $Location*Extension$ and *Operator* were respectively excluded one by one from the reduced Models 2 and 3. The final model (Model 4) included the significant predictors. Chi-square Wald statistics, degrees of freedom and significance for each considered effect in the full, reduced and final models are reported in Table 1.

**Table 2:** Linear regression analysis model for mTRE, reference categories are $\hbox {extension}=\hbox {yes}$ and $\hbox {location}=\hbox {distal}$

Generalized linear model for mTRE	$\upbeta$	95% CI		Wald chi-square	*df*	*P* value
		Lower	Upper			
$\hbox {Extension}=\hbox {no}$	1.361	0.54	2.181	10.562	1	0.001
$\hbox {Location}=\hbox {proximal}$	0.882	$-$ 0.13	1.895	2.917	1	0.088
$\hbox {Location}=\hbox {mid-shaft}$	1.826	0.813	2.838	12.487	1	0.0001

**Fig. 6:**
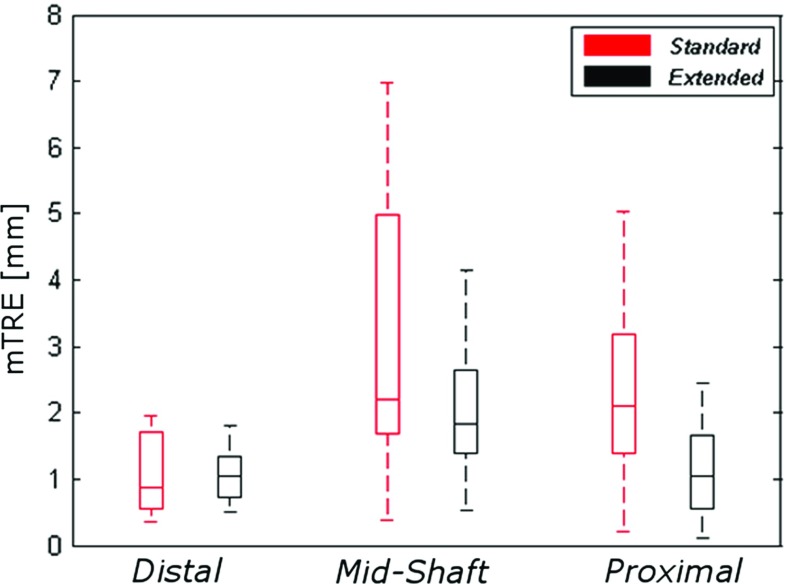
Box plots representing guide positioning error (mTRE) dependency on location and on guide design

Model estimates, p values, confidence intervals and value of Wald Chi-square statistic for the final model are reported in Table 2. A significant relationship was found between the mTRE and mid-shaft guides ($\beta =1.826, p=0.0001$); the positive regression coefficient $\beta$ indicates a significant increase in the positioning error in mid-shaft guides with respect to distal guides. A positive association was also found between mTRE and standard guides ($\beta =1.362, p=0.001$) with respect to extended guides. No significant relationship between the increase in mTRE and proximal guides was found, i.e., it could not be proven that proximal guide positioning was worse than distal guide positioning. Summary statistics (median, IQR) of the mTRE calculated after pooling the data by fitting location and by guide design are reported in Fig. 6.

Guides fitting the distal radius show better positioning accuracy and precision. The guides with extension show an overall improved positioning accuracy and precision.

**Fig. 7:**
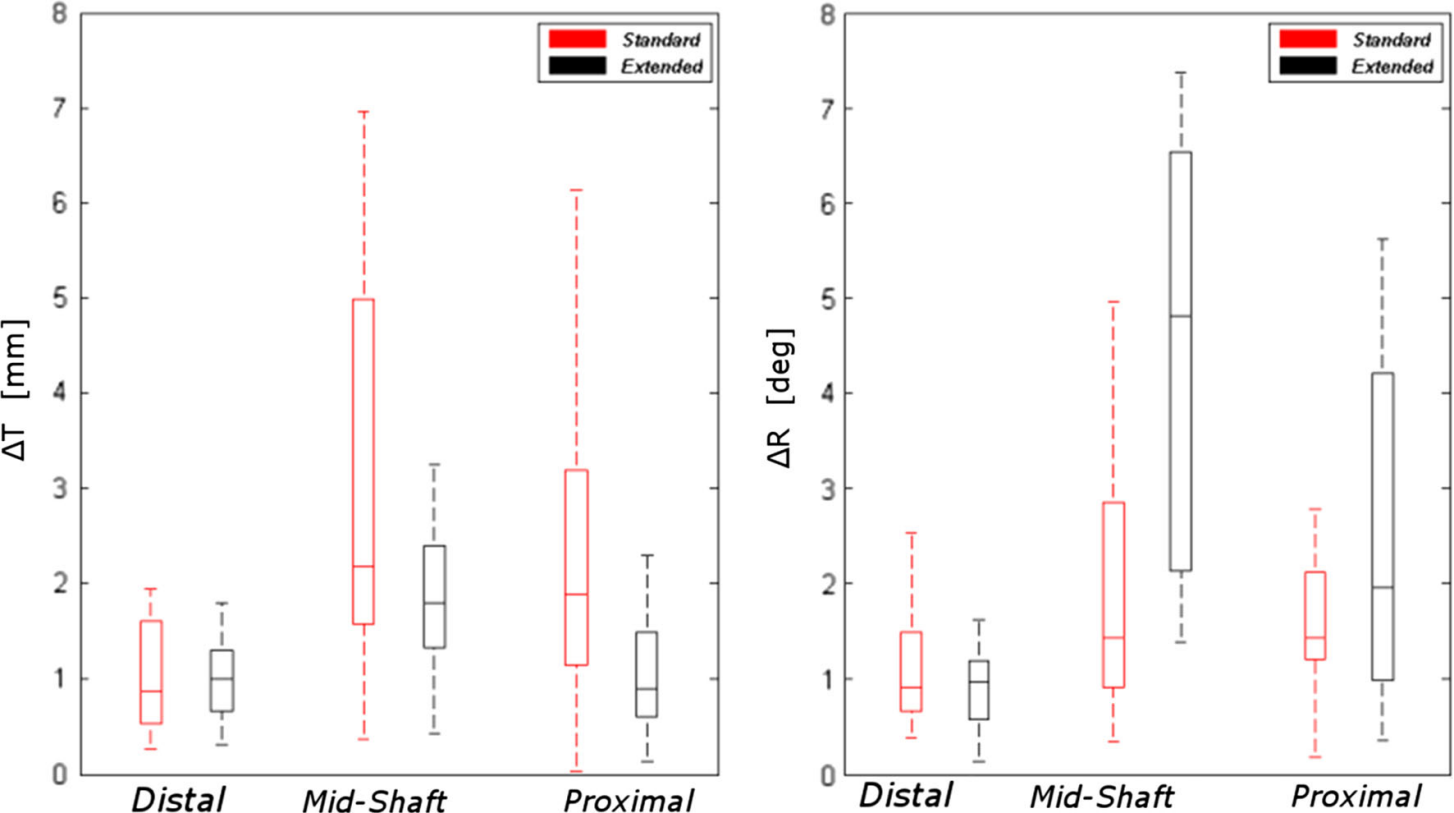
Box plots representing total translation error $\Delta T$ (left) and total rotation error ($\Delta R$) dependency on location and on guide design

Two additional linear regression models were constructed, with the same approach described in the previous paragraph, to evaluate the effect of fitting location and guide design on the total translation ($\Delta {\varvec{T}}$) and rotation ($\Delta {\varvec{R}}$) errors. Chi-square Wald statistics, degrees of freedom and significance for each considered effect in the full, reduced and final models are reported in Tables 3 and 5, in “Appendix”. Positive associations were found between $\Delta {\varvec{T}}$ and mid-shaft guides ($\beta =1.820, p=0.0001$) with respect to distal guides and between $\Delta {\varvec{T}}$ and standard guides ($\beta =1.358, p=0.001$) with respect to extended guides. In the analysis of $\Delta {\varvec{R}},$ a significant relationship was found between $\Delta {\varvec{R}}$ and the guide location, with $\Delta {\varvec{R}}$ being significantly higher in proximal ($\beta =1.536, p=0.007$) and mid-shaft guides ($\beta =1.898, p=0.001$) compared to distal guides.

**Fig. 8:**
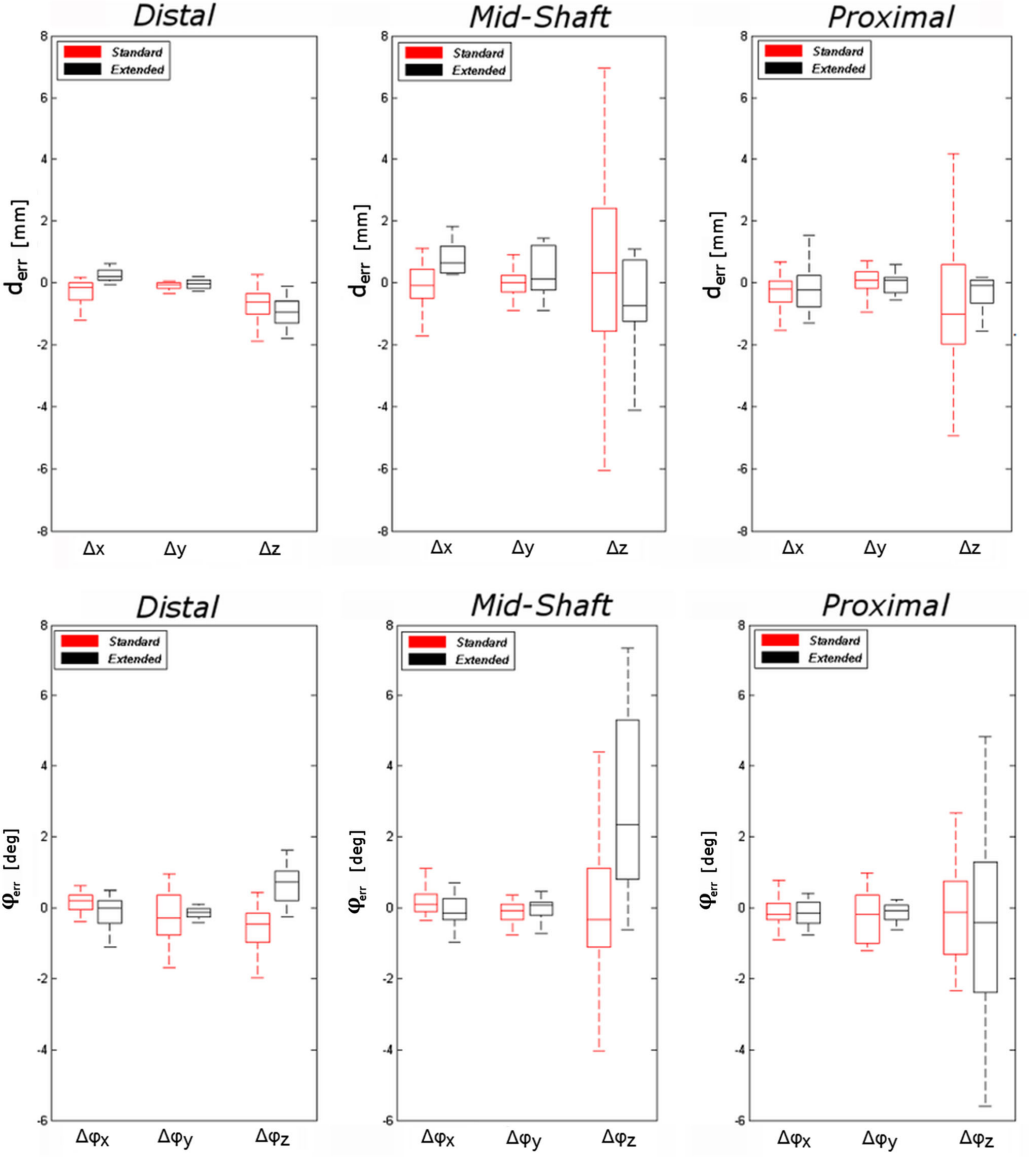
Box plot of guide translational errors (first row) and rotational errors (second row) in six DOF with dependency on location and guide type

The lateral extension of the guides did not significantly affect the proximal and distal guide rotational accuracy, but significantly increased $\Delta R$ ($\beta =2.083, p=0.0001$) in mid-shaft guides. Summary statistics (median, IQR) of $\Delta R$ and $\Delta T$, calculated after pooling the data by fitting location and by guide design, are reported in Fig. 7.

Box plot representing summary statistics of the three translation parameters ($\varDelta x, \varDelta y, \varDelta z$) and three rotation parameters ($\varDelta \varphi x, \varDelta \varphi y, \varDelta \varphi z$) for each guide location (distal, mid-shaft and proximal) and for different guide designs are reported, respectively, in Fig. 8. In distal guides, the variability in translation along the three axis and rotation about the *z*-axis was lower than for mid-shaft and proximal guides. The main effect of the guide extension can be observed in the reduced variability of guide translation along the three directions, especially along *z*-axis direction. Variability of rotation along the *y*-axis was lower in extended guides, but higher along the *z*-axis compared to standard guides.

## Discussion

3D-printed patient-specific orthopedic guides are a promising surgical navigation technique, which is expected to become even more popular and routinely applicable in the next decade due to the rapid developments in 3D printing technology. Many reports have already described encouraging results from the use of customized 3D-printed guides in corrective osteotomy of the radius. Among the most relevant studies, Murase et al. [21, 22], Dobbe et al. [12], Miyake et al. [23], Honigmann et al. [11], Kunz et al. [13], Stockman et al. [24] and Schweizer et al. [25] used patient-specific drilling and cutting guides for the navigation of distal radius corrective osteotomies. Imai et al. [26] reported the accuracy of using custom guides in the correction of a congenital disorder of the radial physis. Finally, Ma et al. [9] have compared the accuracy and precision of using custom guides with image-based navigation techniques for corrective osteotomy of the distal radius in a simulation study. However, in all the aforementioned studies, the outcome of the navigated surgery, which involves many other steps than guide positioning and not the sole positioning accuracy of the 3D-printed guides, has been reported. Moreover, as also recently published reviews on 3D printing in medical setting point out, the majority of these publications largely focus on individual cases, with variable reporting of technical specifications (e.g., CT slice thickness, guide dimensions, computer-aided design software (CAD), printer type, printing material) [4, 5]. This makes it still difficult to come to a conclusive statement regarding the accuracy of 3D-printed surgical cutting guides, despite the large number of publications on the topic [1, 4, 5, 11, 13, 27].

In the current study, we quantitatively investigated the positioning errors in fitting two patient-specific guide designs onto three different fitting locations on the radius surface. The intrinsic accuracy and precision of printing and position evaluation was better than that of guide positioning, which validates our measurement technique. The accuracy that we found for positioning volar distal radius guides is comparable with the findings of Oka et al. [16] who reported the error in setting the location of a customized template on the dorsal side of the distal radius. In their study, they used the Lister’s tubercle as surface anchor for a cutting block of size $50\times 40\times 15\,\hbox {mm}$ and reported an error of less than 1.0 mm and $1.0{^{\circ }}$. As stated by the authors, the dorsal surface of the distal radius, which has quite a characteristic configuration, is not a representative location of placing a guide, because the bone does not always have such a particular shape [16]. Therefore, this last result cannot be generalized for different anatomic regions of the radius and for different guide dimensions. The reported precision and accuracy of volar distal radius guides are also consistent with the findings of Omori et al. [28], who investigated the positioning accuracy of a patient-specific guide for the volar distal radius in the presence of soft tissues in a cadaver experiment.

Our study showed that the mTRE of fitting guides in the volar mid-shaft region, featuring fewer surface anchors and a near-cylindrical shape, was significantly higher ($p=0.0001$), i.e., fitting worse, than the mTRE of fitting guides in the distal volar region. Extended guides significantly improved the overall positioning accuracy by reducing the mTRE in all the considered guide locations ($p=0.001$).

In general, the mTRE is an error metric which is dependent on the shape and scale of the considered objects. However, in this study, comparison between mTRE of fitting different guides was possible because the portions of the guides considered for mTRE computation were very similar in shape and size. Moreover, shape- and size-invariant parameters such as the total translation and the total rotation errors are reported. While the total translation error was significantly reduced for the extended guides in all the regions ($p=0.001$), the total rotation error increased in the mid-shaft extended guides ($p=0.0001$). This last result indicates that the extension of the guide may still not be sufficient to increase the accuracy in the mid-shaft region and could even introduce additional positioning challenge. Considering all six DOF parameters, the main effect of the guide extension can be seen in an increased translation precision along the *z*-axis and an increased rotation precision about the *y*-axis.

In case of cutting, drilling and reduction guides, the reported positioning error can be directly related to drilling, cutting and bone repositioning errors. However, the total error of bone repositioning in corrective osteotomy surgery may be affected by bending of drills or saw-blades as well. Additional positioning errors can be inferred by sub-optimal (pre-)bending of osteosynthesis material [8].

The CT-based technique used to measure the positioning error was accurate and reproducible (translation error ($\hbox {mean}\pm \hbox {SD}) < 0.002\,\pm \,0.01\,\hbox {mm}$ and a rotation error ($\hbox {mean}\pm \hbox {SD}) < 0.013\,\pm \,0.010{^{\circ }}$). Dobbe et al. already reported regarding the accuracy of a similar technique used for preoperative registration of cadaver CT images with less accurate results (translation error ($\hbox {mean}\pm \hbox {SD}) < 0.36\,\pm \,0.13\,\hbox {mm}$ and a rotation error ($\hbox {mean}\pm \hbox {SD}) < 0.12\,\pm \,0.07{^{\circ }}$) [7]. Differences in results can be explained by the fact that the point-to-image registration technique used is directly affected by noise in the image. CT images of human wrists are often affected by a poor boundary contrast due to non-uniform characterization of bone tissue and by the narrow spacing between bones [29]. The improved methodological accuracy observed in this paper may be due to the fact that we used CT images of isolated plastic 3D-printed models with homogenous density, resulting in CT images where the boundaries of the models were sharp and well defined. The registration and segmentation techniques used in this study were very accurate; however, in general, the quality of image segmentation can introduce errors in the guide design and can therefore limit the guide fitting.

In this article, we used simple guide designs comparable with previously published studies [14, 15, 27]. Depending on the surgical target, a guide sometimes requires an adaptation of the design in order to fit around vital soft tissue structures while guaranteeing an adequate fit. The positioning accuracy of a given guide will therefore depend on the chosen design. In this study, we focused on the positioning accuracy of guide placement for only the radius, which can be considered a limitation of our study since different results may be found for different bone types.

## Conclusion

Our study showed that the positioning error of patient-specific cutting and drilling guides depends on the fitting location. This should be carefully taken into account when considering 3D-printed patient-specific guide technology in surgery of the mid-shaft. We recommend using extended guides for future utilization in distal and proximal radius regions since it increases the accuracy and precision of surgical navigation.

## Appendix

**Table 3:** Summary of model effects in the exploratory models tested. Model 4 is the final model for $\Delta T$

	$\Delta T$ Model effects	$\chi ^{2}$	*df*	*P* Value
Model 1	*Extension*	10.530	1	0.001
	*Location*	12.442	2	0.002
	*Location*Extension*	3.513	2	0.173
	*Operator*	6.868	3	0.076
	*Geometry*	5.0	5	0.416
Model 2	*Extension*	10.701	1	0.001
	*Location*	12.684	2	0.002
	*Location*Extension*	3.075	2	0.215
	*Operator*	6.631	3	0.085
Model 3	*Extension*	10.994	1	0.001
	*Location*	13.070	2	0.001
	*Operator*	6.489	3	0.090
Model 4	*Location*	10.507	2	0.001
	*Extension*	12.491	2	0.002

**Table 4:** Linear regression analysis model for $\Delta T$, reference categories are $\hbox {extension}=\hbox {yes}$ and $\hbox {location}=\hbox {distal}$

Generalized linear model for $\Delta T$	$\upbeta$	95% CI		Wald $\chi ^2$	*df*	*P* value
		Lower	Upper			
$\hbox {Extension}=\hbox {no}$	1.358	0.537	2.180	10.507	1	0.001
$\hbox {Location}=\hbox {proximal}$	0.812	$-$ 0.202	1.825	2.465	1	0.116
$\hbox {Location}=\hbox {mid-shaft}$	1.820	0.807	2.834	12.392	1	0.0001

**Table 5:** Summary of model effects in the exploratory models tested

	$\Delta T$ Model effects	$\chi ^{2}$	*df*	*P* value
Model 1	*Extension*	2.675	1	0.102
	*Location*	33.216	2	0.0001
	*Location*Extension*	13.083	2	0.001
	*Operator*	5.668	3	0.129
	*Geometry*	5.895	5	0.317
Model 2	*Extension*	2.870	1	0.090
	*Location*	30.972	2	0.0001
	*Location*Extension*	12.054	2	0.002
	*Operator*	5.439	3	0.142
Model 3	*Extension*	2.763	1	0.096
	*Location*	29.814	2	0.0001
	*Location*Extension*	11.603	2	0.003
Model 4	*Location*	29.814	2	0.0001
	*Location*Extension*	14.721	3	0.002

Model 4 is the final model for $\Delta R$

**Table 6:** Linear regression analysis model for $\Delta R$, reference categories are $\hbox {extension}=\hbox {yes}$ and $\hbox {location}=\hbox {distal}$

Generalized linear model for mTRE	$\upbeta$	95% CI		Wald $\chi ^2$	*df*	*P* value
		Lower	Upper			
$\hbox {Location}=\hbox {proximal}$	1.536	0.412	2.660	7.177	1	0.007
$\hbox {Location}=\hbox {mid-shaft}$	1.898	0.827	2.970	12.058	1	0.001
($\hbox {Extension}=\hbox {no}$)*($\hbox {Location}=\hbox {distal}$)	0.513	$-$ 0.611	1.636	0.799	1	0.371
($\hbox {Extension}=\hbox {no}$)*($\hbox {Location}=\hbox {mid-shaft}$)	$-$ 2.083	$-$ 3.110	$-$ 0.967	13.904	1	0.000
($\hbox {Extension}=\hbox {no}$)*($\hbox {Location}=\hbox {proximal}$)	$-$ 0.074	$-$ 1.146	0.997	0.018	1	0.892

Details on the generalized linear regression models constructed for the total translation error $\Delta T$ and total rotation error $\Delta R$ are reported in the following Tables. Chi-square Wald statistics, degrees of freedom and significance for each considered effect in the full, reduced and final models for $\Delta T$ and $\Delta R$ are respectively reported in Tables 3 and 4. Model estimates, *p* values, confidence intervals and value of Wald Chi-square statistic for the final models of $\Delta T$ and $\Delta R$ are respectively reported in Tables 5 and 6.
